# *Commiphora gileadensis* ameliorate infertility and erectile dysfunction in diabetic male mice

**DOI:** 10.1515/med-2025-1166

**Published:** 2025-03-10

**Authors:** Ayman Saeed Alhazmi

**Affiliations:** Department of Clinical Laboratory Sciences, College of Applied Medical Sciences, Taif University, Taif, 24227, 20006 Saudi Arabia

**Keywords:** *Commiphora gileadensis*, infertility, erectile dysfunction, adropin

## Abstract

**Background:**

The *Commiphora gileadensis* (*C. gileadensis*) is a tree belonging to the genus *Commiphora.* Aim of the study: This study investigates the effect of *C. gileadensis* on erectile dysfunction and infertility in male mice.

**Methods:**

Fifty male BALB/c mice are divided into five groups: control, untreated diabetic, diabetic *C. gileadensis* sap-treated, methanol extract-treated, and acetone extract-treated. All groups were assessed for body weight, testicular weight, serum follicle-stimulating hormone, luteinizing hormone, testosterone, prolactin, nitric oxide, adropin, endothelin levels, semen analysis, CD4^+^, CD8^+^, CD25^+^, and testicular nitric oxide synthase (NOS) immunoreactivity.

**Outcome:**

*C. gileade*nsis maintains sexual integrity and infertility in mice.

**Results:**

Diabetic groups treated with *C. gileadensis* had significantly higher body weight than the untreated group. Furthermore, the diabetic group treated with *C. gileadensis* sap had significantly increased testicular weight than the untreated groups. Diabetic groups treated with *C. gileadensis* had significantly greater testosterone levels than the untreated groups. Additionally, these groups exhibit considerably higher nitric oxide and adropin levels than the untreated diabetic group. Endothelin levels were considerably lower in diabetic groups treated with *C. gileadensis* than in the untreated group. Semen analysis shows that the diabetic group treated with *C. gileadensis* sap had considerably more sperm count than the untreated group (*P* < 0.05). CD4^+^, CD8^+^, CD4^+^, CD25^+^, and CD8^+^ CD25^+^ were reduced significantly in diabetic mice treated with *C. gileadnesis*. In addition, the NOS immunoreactivity is greater in diabetic *C. gileadensis* treated groups than in the untreated group.

**Clinical implications:**

*C. gileadensis* induces mice erectile function and fertility.

**Strength and limitations:**

The study does not use laser Doppler flowmetry for the measurement of erectile dysfunction.

**Conclusion:**

*C. gileadensis* ameliorates infertility and erectile dysfunction in diabetic mice.

## 1 Introduction

A significant portion of the population is afflicted by the crippling conditions of infertility and sexual dysfunction. Infertility has been classified as a public health concern by the WHO. According to studies by Gabr et al. and McCabe et al., erectile dysfunction and premature ejaculation are prevalent among men of reproductive age, with the prevalence ranging from 8 to 31% [1,2]. Infertility is defined as 1 year of regular, unprotected sexual activity without conception. Sexual dysfunction is the primary cause of male infertility [3–5]. Organic factors such as cardiovascular, metabolic, neurogenic, and endocrine disorders; relational factors such as family and marital disagreements; and intrapsychic disruptions such as anxiety and depression can all lead to erectile dysfunction [6]. Additionally, erectile dysfunction in men with infertility is an independent risk factor for a decline in sexual engagement and negatively impacts fertility [7].

Erectile dysfunction can be treated through changes in lifestyle, oral and parenteral medications, injectable vasodilator drugs, vacuum erection devices, surgery, and psychosexual therapy either with the patient alone or with the involvement of the patient’s partner. The frequency of erectile dysfunction occurrence in male infertility has not been extensively studied by experts. In a small cohort study utilizing a nonvalidated approach involving 16 couples, reported that 11 males claimed to experience impotence upon discovering their infertility [5].

The primary chemical mediator of smooth muscle relaxation involved in penile erection is nitric oxide. In the penile corpora cavernosa, neuronal and endothelial cells release nitric oxide [8]. In general, endothelin is employed as a measure of endothelial dysfunction and is a modulator of penile erection due to its powerful vasoconstrictor properties [9]. The endothelium tissue expresses the protein adropin [10]. By overexpressing nitric oxide synthases (NOS), adropin plays a crucial role in endothelium protection [11].

The Arabian balsam tree known as *Commiphora gileadensis* is a member of the genus *Commiphora* and is indigenous to the Arabian Peninsula and southern Egypt. The tree’s sap, wood, bark, and seeds have significant medicinal properties [12]. The tree is used in traditional Arabian medicine to treat inflammatory conditions, constipation, stomachaches, joint discomfort, and headaches. Numerous studies have noted the tree’s antimicrobial capabilities; however, no studies have examined how it affects mice’s infertility and erectile dysfunction [13]. Moreover, the sap of *C. gileadensis* has been used as an antibacterial agent in both *in vivo* and *in vitro* studies [14]. Furthermore, a previous study found that the methanolic extract of *C. gileadensis* has antibacterial effects and aids in wound healing [15]. This study aimed to investigate the effect of different extracts of *C. gileadensis* on male mice infertility and erectile dysfunction.

## 2 Materials and methods

This analytical study was carried out at the Faculty of Applied Medical Sciences, Taif University, between June 2023 and August 2023.

### 2.1 *C. gileadensis* collection

*C. gileadensis* was collected from a high mountain area called the Alaab Valley, located near the Al-Madinah region of western Saudi Arabia. Leaves and fallen branches of the tree were collected in October 2022.

### 2.2 Preparation of *C. gileadensis* sap

The apical portions of *C. gileadensis* branches were pruned, leaving a 5 mm distance from the ends, and the exuding sap was promptly collected following the incision. After being mixed with an equal volume of ethanol, the sap was subjected to centrifugation at a speed of 10,000 rpm for a duration of 10 min, subsequent to agitation for 15 min at ambient temperature. Subsequently, the supernatant was stored at a temperature of −20°C until it was subjected to analysis [16].

### 2.3 Preparation of *C. gileadensis* methanolic extract

The leaves and branches of *C. gileadensis* were subjected to a cleaning process using tap water, followed by drying in a hot-air oven at a temperature of 40°C. After undergoing the drying process, the substance was further transformed into a finely ground form and subjected to sieving in order to eliminate any significant impurities. Subsequently, a quantity of 10 g of the aforementioned powder was subjected to maceration within a sterile funnel for a duration of 24 h, utilizing a solvent volume of 100 mL consisting entirely of methanol with a purity of 100%. The funnel was aggressively agitated prior to the filtration process, which involved the use of sterile filter paper. The *C. gileadensis* extract obtained was subjected to drying in a water bath at a temperature of 40°C in order to get a concentrated extract. The sample was thereafter refrigerated at a temperature of 4°C for a duration of 2 weeks and afterward transferred to a storage temperature of −20°C for future analysis [17].

### 2.4 Preparation of *C. gileadensis* acetone extract

The leaves and branches of *C. gileadensis* were subjected to a drying process at a temperature of 60°C for a duration of 6 h in a vacuum oven. Subsequently, the dried plant material was meticulously chopped into minute fragments using a razor blade, resulting in the formation of a powdered substance. Subsequently, a quantity of 10 g of the unprocessed *C. gileadensis* fragments were submerged in a solution consisting of 200 mL of acetone for a duration of 3 days, maintaining the ambient temperature. Throughout this time frame, the acetone solution, which was homogenized using a magnetic stirrer, was renewed on a daily basis. The sample obtained from the extraction process was subjected to drying using a rotary evaporator in order to eliminate any remaining traces of acetone. Subsequently, the sample was kept at a temperature of −20°C in preparation for subsequent analysis [18].

### 2.5 Characterization of using ultraperformance liquid chromatography coupled with mass spectrometer (UPLC–MS)

Samples were thawed on ice, adding 1.5 mL chloroform:methanol (2:1, v/v), 0.5 mL ultrapure water, vortexed for 1 min, then centrifuged for 10 min at 3,000 rpm at 4°C. Transfer the lower phase to a fresh tube and dry under nitrogen. For lipidomic analysis, the dried extract was resuspended in 200 µL of isopropyl alcohol: MeOH (1:1, v/v) and added 5 µL of 1-heptadecanoyl-2-hydroxy-sn-glycero-3-phosphocholine LPC (12:0) as internal standards After centrifuging for 10 min at 12,000 rpm at 4°C, transfer the supernatant for LC-MS analysis. Separation is done using Ultimate 3000 LC and Q Exactive MS (Thermo, Waltham, MA, USA), followed by ESI-MS screening. The LC system uses ACQUITY UPLC BEH C18 (100 mm × 2.1 mm × 1.7 mm) and Ultimate 3000 LC. The mobile phase consists of solvents A (60% acetonitrile + 40% H_2_O + 10 mM ammonium formate) and B (10% acetonitrile + 90% isopropyl alcohol + 10 mM ammonium formate) with gradient elution (0–100% B, 30–100% B, 10.5–12.5% B, 100% B, 12.5–12.51 min, 100–30% B, 12.51–16.0 min, 30% B). A 0.3 mL/min flow rate is used for the mobile phase. Column temperature is fixed at 40°C, whereas sample manager temperature is set at 4°C. The following parameters are specified for ESI negative mode mass spectrometry: ESI−: heater temp 300°C, sheath flow rate 45 arb, aux flow rate 15 arb, sweep flow rate 1 arb; spray voltage 3.2 kV; capillary temp 350°C; S-Lens RF level 60% [15].

### 2.6 Experiment design

Fifty male BALB/c mice, aged 2 months and weighing 20–25 g, were obtained from the Umm Al-Qura University animal house. The mice were housed in a standard rodent cage with woodchip bedding. The cage was placed in a large, well-ventilated room with a 12 h light/dark cycle and maintained at a temperature of 25°C. A standard rodent diet and tap water were provided to all the mice throughout the experiment’s duration. After 2 weeks of acclimatization, 50 mice were randomly divided into five groups with ten mice each.

1. The first group served as the negative control and received no treatment.
2. The second group was the positive control (diabetic group). The mice in this group were injected with 55 mg/kg body weight of streptozotocin intraperitoneally for 5 consecutive days to induce diabetes mellitus.
3. The third group was designated as the *C. gileadensis* sap-treated (CGS) group. After the induction of diabetes mellitus, all the mice in this group were treated with a *C. gileadensis* sap dose of 200 mg/kg per day via intragastric gavage after 1 week of streptozotocin administration [15].
4. The fourth group was the *C. gileadensis* methanolic extract-treated (CGM) group. Similar to the second group, diabetes mellitus was induced in the mice in this group. In addition, they received 200 mg/kg body weight per day of *C. gileadensis* methanolic extract via intragastric gavage after 1 week of streptozotocin administration.
5. The fifth group was the *C. gileadensis* acetone extract-treated (CGA) group. Diabetes mellitus was induced in mice in this group as the second group. In addition, they received 200 mg/kg per day of *C. gileadensis* acetone extract via intragastric gavage following the administration of streptozotocin.

### 2.7 Body weight measurement

The body weight of each mouse included in this study was measured at the end of the experiment using a digital balance (OHAUS, Scout Pro SPU601, China).

### 2.8 Blood collection

Blood samples were collected for all 50 mice from the retro-orbital venous plexus in plain tubes after 5 days of streptozotocin administration for blood glucose estimation and at the end of the experiment (after 4 months) for biochemical markers measurement. All blood samples were immediately centrifuged at 2,500 rpm for 15 min, and the resulting blood sera were stored at −80°C for further analysis.

### 2.9 Preparation of testicular tissue

After 4 months, all the mice were euthanized. Their testes were rapidly excised for testicular weight measurement, testicular homogenate preparation, and immunohistochemistry analysis. The testes of each mouse were weighed and the ratio of testes weight to body weight was calculated. Semen was ground from the epididymis and incubated for 15 min in a sterile Petri dish containing 0.9 mL of RPMI-1640 medium.

### 2.10 Preparation of testicular homogenate

Using an OMNI International Inc. 2 mL bead kit, a testicular homogenate was prepared. A protease inhibitor, radio-immunoprecipitation assay buffer, and small pieces of testis were added to a 2 mL microtube containing beads. The microtube was put in a homogenizer for 2 min (Bead Ruptor 12, OMNI International Inc.), then centrifuged for 30 min at 8°C and 15,000 rpm (SIGMA 1-14 k). Until analysis, the supernatant was kept at −20°C after being aspirated into a microtube and centrifuged for 15 min at 8°C and 15,000 rpm [19].

### 2.11 Estimation of testicular homogenate antioxidant and oxidative stress parameters

Lipid peroxide (LPO), reduced glutathione (GSH), glutathione peroxidase (GSH-Px), superoxide dismutase (SOD), and catalase (Elabscience kits, USA) were estimated by colorimetric assay using Varioskan LUX (Thermo Fisher Scientific, USA).

### 2.12 Semen analysis

#### 2.12.1 Sperm count

Ten microliters of diluted sperm suspension were placed in the counting chamber of a hemocytometer. Under a light microscope, counting began at 200× magnification after a 5 min incubation period [20].

#### 2.12.2 Sperm morphology

A 40 L sample of sperm suspension was incubated with 10 L of 1% eosin and nigrosine stain in a test tube for 1 h at room temperature. Then, one drop of the suspension was applied to a slide and examined at 400× magnification. For each mouse, a total of 200 sperms were examined [21].

#### 2.12.3 Sperm motility

A drop of sperm suspension was examined at 1,000× magnification under a light microscope to assess its motility. The percentage of motile sperm was calculated using the following formula: percentage of dead sperms = (number of dead sperms × 100)/total number of sperms examined [22].

### 2.13 Measurement of serum parameters

Serum testosterone, follicle-stimulating hormone (FSH), luteinizing hormone (LH), and prolactin were measured using the quantitative competitive ELISA method and the Varioskan TM LUX instrument (Thermo Fisher Scientific, USA) [23]. In addition, serum endothelin, adropin, and nitric oxide levels were measured using the same method and instrument [24]. Samples used for the estimation of testosterone levels for diabetic *C. gileadensis* sap-treated mice were diluted because the undiluted testosterone level samples were out of the range of the bio-machine.

### 2.14 Estimation of CD3^+^, CD4^+^, CD8^+^, and CD25^+^

EDTA tube blood samples were carefully deposited on Ficoll-Paque density gradient medium in a 15 mL conical tube. Continuous 400×*g* centrifugation at 4°C for 30 min was performed on the samples. The mononuclear cell layer was carefully removed and placed in a new tube. PBS with 2% fetal bovine serum and 0.1% sodium azide was used to stain the cells after two PBS washes. The cell suspension was tagged with fluorochrome-conjugated monoclonal antibodies (CD3^+^, CD4^+^, CD8^+^, and CD25^+^) in four tubes of 100 μL each. The staining took 30 min at 4°C in the dark. After staining, the cells were washed with buffer and fixed with 1% paraformaldehyde. At 4°C, all samples were incubated for 30 min without light. After washing, the samples were resuspended in flow cytometry sample preparation buffer. Next, the materials were flow cytometer-analyzed [25].

### 2.15 Testicular immunohistochemistry analysis

The mice were euthanized and dissected, and their testes were quickly removed for immunohistochemistry evaluation of NOS. The testicular tissue samples were fixed in 10% buffered formalin for at least 24 h before being transferred to 70% ethanol. The tissues were then embedded in paraffin blocks, and sections (slides) of approximately 5 μm were prepared. The sections were placed in xylene for 5 min and rehydrated in 100 and 95% ethanol in that order for 10 min and heated for a further 10 min in a microwave oven before being cooled for 30 min. The slides were then washed three times for 5 min in distilled water and stained with NOS antibodies. Finally, the slides were washed three times for 5 min with a wash buffer. Under a light microscope, the slides were examined qualitatively using the Sulzbacher scoring system.

### 2.16 Statistical analysis

Statistical analysis was performed using SPSS software version 16 (SPSS Inc., Chicago, IL, USA). All data were expressed as mean ± SD, and all comparisons of total chemical parameters included in this project between different groups were done through one-way analysis of variance. The level of significance was set at *P* < 0.05.


**Ethical approval:** The animal study protocol was accredited by the National Committee for Bioethics at Taif University (protocol code HAO-02-T-105) and the Committee considered that the proposal fulfills the requirements.

## 3 Results

### 3.1 LC–MS of *C. gileadensis* extracts

The LC–MS chromatographic profile in negative mode showed several lipid components. Ceramide 69%, hexosylceramide 18%, and phosphatidylethanolamine 7.6% were found to be the most abundant lipid components. Moreover, the extracts showed high quantities of steroids.

### 3.2 Blood glucose levels

After 5 days of streptozotocin injection, random blood glucose levels were found to be elevated in all diabetic groups. The random blood glucose levels ranged 380–420 mg/dL for all mice in the five groups.

### 3.3 Body and testicular weights

Table 1 presents the body weight, testicular weight, and testicle/body weight ratio of all groups after 4 months. Over the course of 16 weeks, the weight of the CGS (32.11 ± 4.31), CGM (32.55 ± 3.06), and CGA (30.31 ± 3.33) groups had significantly higher body weights compared with the untreated diabetic mice (24.14 ± 1.33) (*P* < 0.05). Furthermore, the testicular weight of the CGS group (1.87 ± 0.07) was significantly higher compared with that of the diabetic untreated group (1.10 ± 0.07) (*P* < 0.05).

**Table 1:** Bodyweight, testicular weight, and testis body weight ratio of all groups

	Group 1 control (*n* = 10)	Group 2 DM (*n* = 10)	Group 3 DM + CGS (*n* = 10)	Group 4 DM + CGM (*n* = 10)	Group 5 DM + CGA (*n* = 10)
Body weight (g)	32.48 ± 4.25	24.14 ± 1.33	32.11 ± 4.31*	32.55 ± 3.06*	30.31 ± 3.33*
Testis weight (g)	1.20 ± 0.08	1.10 ± 0.07	1.87 ± 0.07*	1.35 ± 0.07	1.31 ± 0.06
Testicular weight/body weight ratio	0.040 ± 0.003	0.046 ± 0.002	0.058 ± 0.001	0.041 ± 0.002	0.043 ± 0.003

*P < 0.05, **P < 0.01; *C. gileadensis* sap treated (CGS), *C. gileadensis* methanol treated (CGM), *C. gileadensis* acetone treated (CGA), diabetes mellitus (DM).

### 3.4 Oxidative stress and antioxidant parameters

The concentration of LPO was reduced in CGS (12.44 ± 3.18), CGM (17.22 ± 1.76), and CGA (16.49 ± 2.11) groups compared to untreated diabetic mice (43.61 ± 9.44) (*P* < 0.05). Reduced GSH levels in the CGS (8.16 ± 0.22), CGM (7.77 ± 0.39), and CGA (7.52 ± 1.36) were significantly higher than in the untreated diabetic group (1.81 ± 0.010) (*P* < 0.05). Moreover, the GSH-Px activity was markedly induced in CGS (2.62 ± 0.10) and CGM (1.55 ± 0.02) groups compared to the diabetic untreated mice (0.31 ± 0.03) (*P* < 0.05). The CGS group had significantly higher SOD enzyme activity than the untreated diabetic group (32.44 ± 3.01 vs 15.031 ± 3.96, respectively) (*P* < 0.05) (Table 2).

**Table 2:** Lipid peroxidation, reduced GSH, catalase, GSH-Px, and SOD in testis of all groups

	Group 1 control (*n* = 10)	Group 2 DM (*n* = 10)	Group 3 DM + CGS (*n* = 10)	Group 4 DM + CGM (*n* = 10)	Group 5 DM + CGA (*n* = 10)
LPO (nmol/g)	24.55 ± 4.08	43.61 ± 9.44	12.44 ± 3.18*	17.22 ± 1.76*	16.49 ± 2.11*
GSH (nmol/g)	4.80 ± 1.01	1.81 ± 0.010	8.16 ± 0.22*	7.77 ± 0.39*	7.52 ± 1.36*
Catalase (U/g)	46.02 ± 6.63	12.71 ± 6.92	22.14 ± 1.74	23.05 ± 3.04	18.84 ± 1.81
GSH-Px (U/g protein)	0.66 ± 0.02	0.31 ± 0.03	2.62 ± 0.10*	1.55 ± 0.02*	0.66 ± 0.02
SOD (µg/g protein)	23.73 ± 5.05	15.031 ± 3.96	32.44 ± 3.01*	18.81 ± 2.26	19.77 ± 2.88

*P < 0.05, **P < 0.01; *C. gileadensis* sap treated (CGS), *C. gileadensis* methanol treated (CGM), *C. gileadensis* acetone treated (CGA), diabetes mellitus (DM).

### 3.5 Biochemical markers and hormone levels

Table 3 displays the biochemical markers of erectile function, LH, FSH, testosterone, and prolactin serum levels. The CGS group had significantly higher testosterone levels (76.018 ± 5.58) compared to all other groups (*P* < 0.0001). Furthermore, CGM (7.53 ± 0.37) and CGA (6.90 ± 0.17) groups had significantly higher testosterone levels compared to the untreated diabetic (0.15 ± 0.01) and control (2.92 ± 0.01) groups (*P* < 0.01). Compared with the untreated diabetic (8.21 ± 1.68) group, the CGS (45.01 ± 6.08), CGM (36.77 ± 3.28), and CGA (31.42 ± 4.53) groups had significantly higher nitric oxide levels (*P* < 0.01). In addition, the CGS group had a significantly higher nitric oxide level compared with the control group (24.03 ± 3.06) (*P* < 0.05). There were statistically significant differences in adropin levels between the CGS (31.02 ± 5.94), CGM (20.44 ± 3.88), and CGA (21.11 ± 2.09) groups and the untreated diabetic group (8.89 ± 2.22) (*P* < 0.01). Endothelin levels in the CGS (12.44 ± 2.40), CGM (13.06 ± 1.07), and CGA (12.86 ± 2.14) groups were significantly lower compared with the untreated diabetic group (43.50 ± 13.61) (*P* < 0.01).

**Table 3:** Male sex hormones, prolactin, and erectile function markers levels in all five groups

	Group 1 control (*n* = 10)	Group 2 DM (*n* = 10)	Group 3 DM + CGS (*n* = 10)	Group 4 DM + CGM (*n* = 10)	Group 5 DM + CGA (*n* = 10)
FSH (ng/mL)	0.026 ± 0.0001	0.09 ± 0.01	0.022 ± 0.002	0.033 ± 0.006	0.024 ± 0.0005
LH (ng/mL)	0.030 ± 0.0005	0.26 ± 0.02	0.041 ± 0.0015	0.029 ± 0.0003	0.025 ± 0.0004
Testosterone (ng/mL)	2.92 ± 0.01	0.15 ± 0.01	76.018 ± 5.58***	7.53 ± 0.37**	6.90 ± 0.17**
Prolactin (ng/mL)	1.919 ± 0.0065	2.21 ± 0.23	1.39 ± 0.0063	1.08 ± 0.02	1.33 ± 0.02
Nitric oxide (pg/mL)	24.03 ± 3.06	8.21 ± 1.68	45.01 ± 6.08**	36.77 ± 3.28**	31.42 ± 4.53**
Adropin (µg/mL)	18.42 ± 4.79	8.89 ± 2.22	31.02 ± 5.94**	20.44 ± 3.88**	21.11 ± 2.09**
Endothelin (µg/mL)	11.12 ± 3.33	43.50 ± 13.61	12.44 ± 2.40**	13.06 ± 1.07**	12.86 ± 2.14**

*P < 0.05, ** *P* < 0.01; *C. gileadensis* sap treated (CGS), *C. gileadensis* methanol treated (CGM), *C. gileadensis* acetone treated (CGA), diabetes mellitus (DM).

### 3.6 Semen analysis

The semen analysis of all five groups is displayed in Table 4. The CGS group had a significantly higher sperm count (111 ± 17) than the untreated diabetic group (67 ± 28) (*P* < 0.05). Despite the absence of statistically significant differences in morphology and motility, the comparison between diabetic mice treated with different *C. gileadensis* extracts and untreated diabetic mice reveal that treated mice had higher values for sperm count, morphology, and motility.

**Table 4:** Semen analysis of all five groups

	Group 1 control (*n* = 10)	Group 2 DM (*n* = 10)	Group 3 DM + CGS (*n* = 10)	Group 4 DM + CGM (*n* = 10)	Group 5 DM + CGA (*n* = 10)
Sperm count (10^6^)	102 ± 13	67 ± 28	111 ± 17*	98 ± 22	77 ± 36
Rapid motile (%)	42 ± 18	27 ± 9	40 ± 6	38 ± 7	32 ± 8
Slow motile (%)	23 ± 5	38 ± 6	30 ± 16	33 ± 14	31 ± 21
Immotile (%)	32 ± 11	37 ± 7	30 ± 9	29 ± 11	33 ± 4
Normal sperm (%)	84 ± 27	52 ± 37	70 ± 26	63 ± 31	52 ± 14
Abnormal sperm (%)	16 ± 4	33 ± 2	18 ± 25	29 ± 8	30 ± 29

*P < 0.05, ** *P* < 0.01; *C. gileadensis* sap treated (CGS), *C. gileadensis* methanol treated (CGM), *C. gileadensis* acetone treated (CGA), diabetes mellitus (DM).

### 3.7 Peripheral CD3^+^, CD4^+^, CD8^+^, and CD25^+^

**Figure 1:**
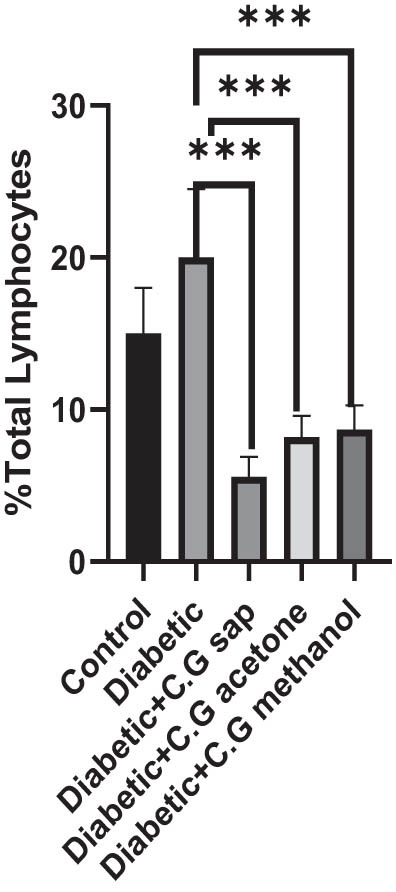
Total lymphocytes in all groups.

**Figure 2:**
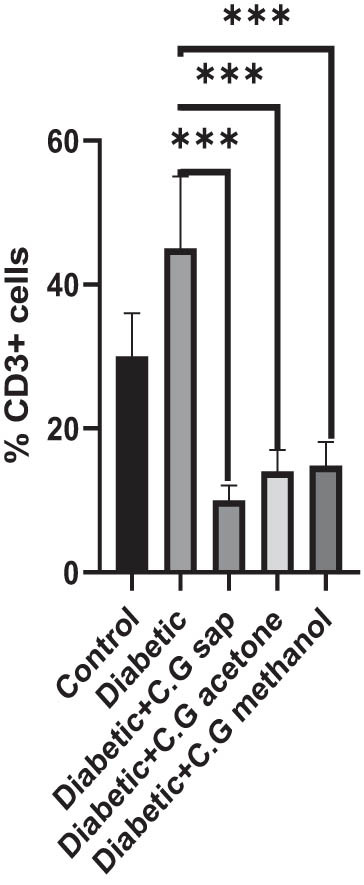
CD3^+^ cells in all groups.

**Figure 3:**
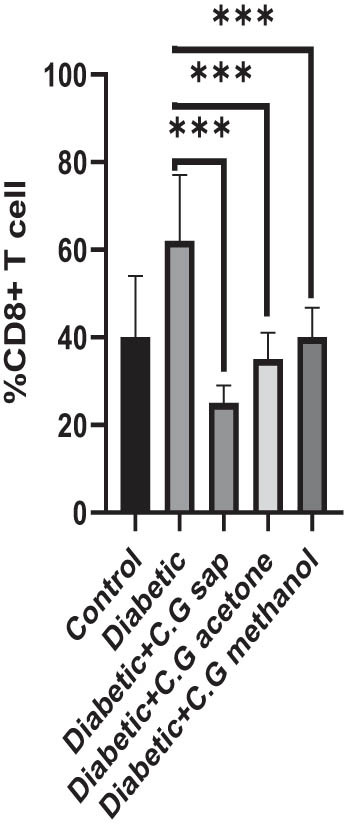
CD4^+^ cells in all groups.

**Figure 4:**
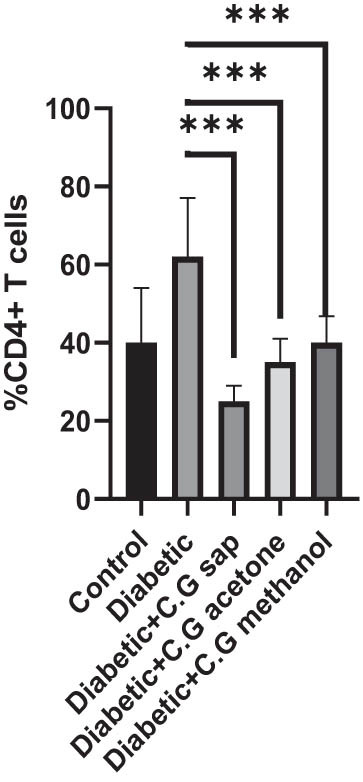
CD8^+^ cells in all groups.

**Figure 5:**
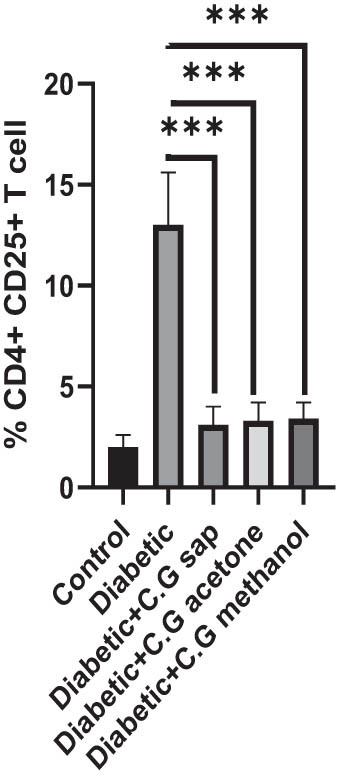
CD4^+^ CD25^+^ cells in all groups.

**Figure 6:**
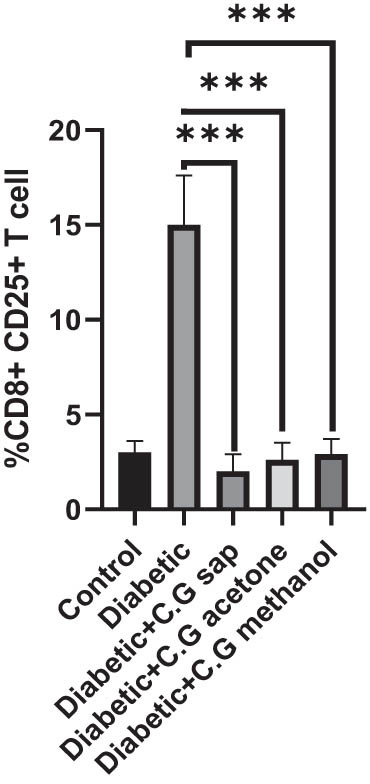
CD8^+^ CD25^+^ cells in all groups.

The CGS, CGM, and CGA showed a substantial decrease in total lymphocytes compared to the untreated diabetic group (*P* < 0.001) (Figure 1). Treatment of diabetic mice with CGS, CGM, and CGA extracts significantly reduced CD3^+^ T cells compared to the untreated group (*P* < 0.001) (Figure 2). In addition, CGS, CGM, and CGA extracts significantly reduced CD8^+^ T cells in diabetic groups (*P* < 0.001) (Figure 3). Results show a significant CD4^+^ reduction in CGS, CGM, and CGA groups compared to diabetic untreated groups (*P* < 0.001) (Figure 4). The CD4^+^ CD25^+^ cell population significantly decreased in all diabetic groups compared to the control group (*P* < 0.001) (Figure 5). Low CD8^+^ CD25^+^ cell counts were seen in CGS, CGM, and CGA groups compared to those not treated (*P* < 0.001) (Figure 6).

### 3.8 NOS immunohistochemistry in testicular tissue

**Figure 7:**
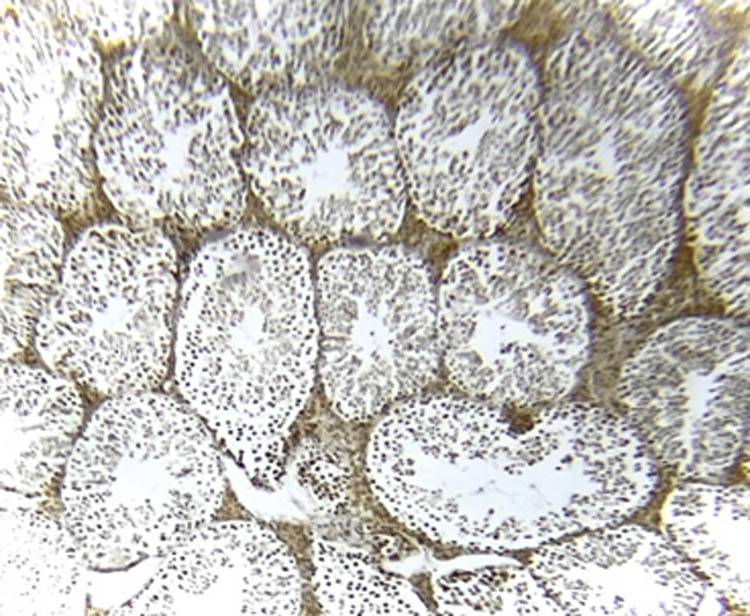
NOS immunoreactivity in the control group.

**Figure 8:**
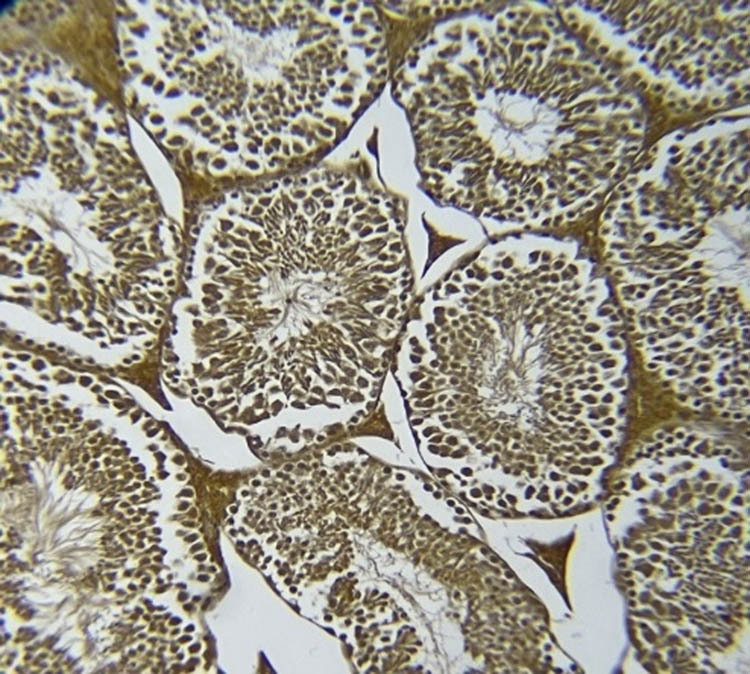
NOS immunoreactivity in the diabetic *C. gileadensis* sap-treated group.

**Figure 9:**
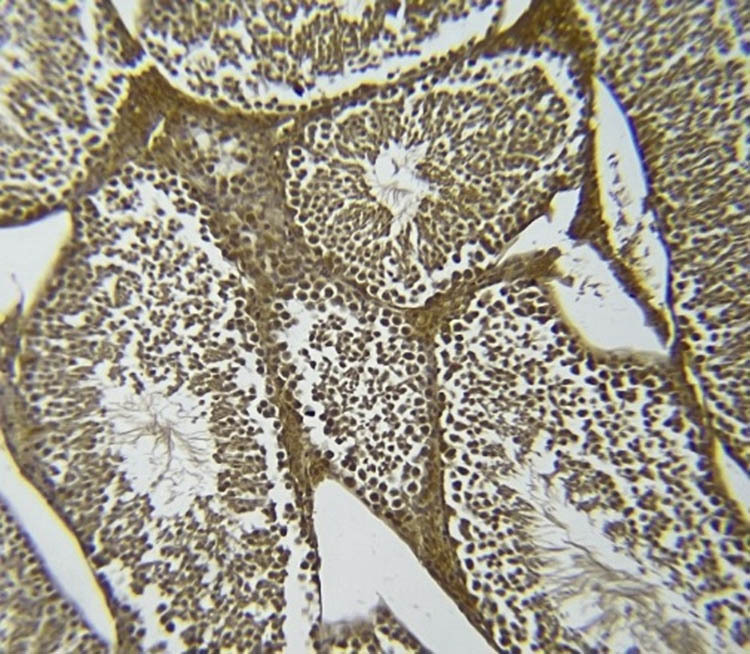
NOS immunoreactivity in the diabetic *C. gileadensis* methanol-treated group.

**Figure 10:**
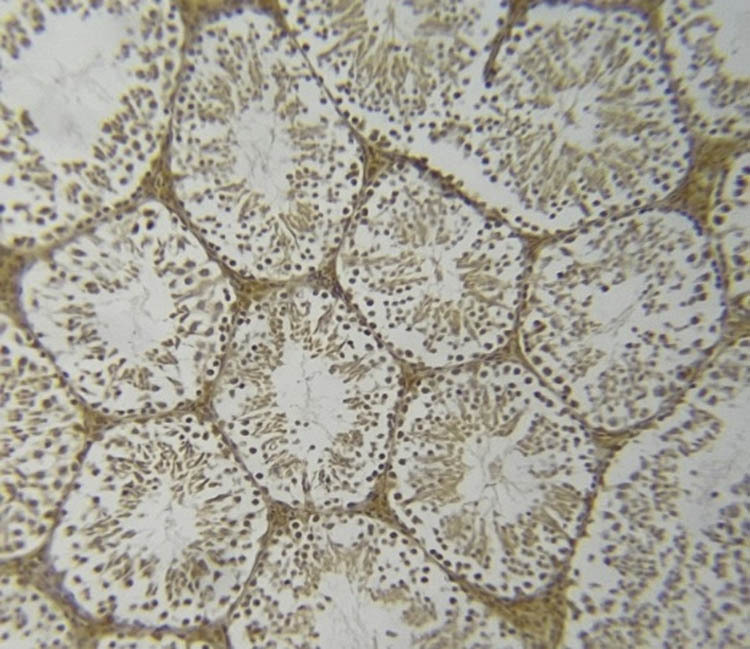
NOS immunoreactivity in the diabetic *C. gileadensis* acetone-treated group.

**Figure 11:**
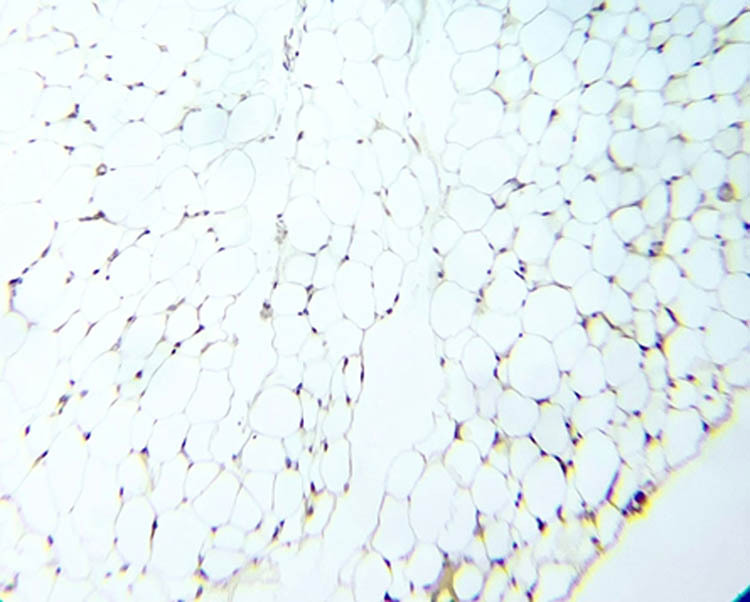
NOS immunoreactivity in the untreated diabetic group.

Figures 7–11 represent the immunoreactivity of NOS in all groups. The immunoreactivity of NOS was significantly greater in mice treated with CGS (Figure 8), CGM (Figure 9), and CGA extracts (Figure 10) than in the control (Figure 7) and untreated diabetic (Figure 11) groups. In the untreated diabetic group, normal testicular architecture (seminiferous tubules) was destroyed and replaced by adipose tissue. Furthermore, the immunoreactivity of the enzyme in the untreated diabetic group was the lowest (Figure 11). Treatment with different *C. gileadensis* extracts restored the normal architecture of testicular tissue in diabetic mice.

## 4 Discussion

The increase in the prevalence of infertility and subfertility has led to an increasing demand for the use of assisted reproduction techniques (ART). Hypertensive disorders of pregnancy and preeclampsia are the most common pregnancy complications and causes of maternal morbidity and mortality. In recent years, the increasing application of ART has resulted in the gradual enhancement of procedures, with embryo freezing being a significant advance. This treatment is specifically provided as a customized method to diminish the occurrence of multiple pregnancies and, crucially, to mitigate the risk of ovarian hyperstimulation syndrome [26]. Two techniques were used frozen embryo fresh (FEF) and frozen embryo transfer (FET). Previously, a study aimed to evaluate the neonatal outcomes in FEF and FET. It found that increased birth weight is shown to be greater in the FET technique, whereas for perinatal morbidity and mortality, the risk appears to be similar between the two groups. For malformation, the studies reported comparable malformation rates between the two groups. Another important finding concerns the incidence of neurodevelopmental abnormalities, with apparently no difference between the two groups. This discovery, along with the latest scientific information concerning the safety of ART on the neuro-psychomotor outcomes of neonates, is significant and reassuring for all couples facing infertility and sub-fertility issues [27]. The present study directed to use of natural products to ameliorate infertility and impotence in males. It uses a number of erection markers and sex hormones to evaluate the effect of *C. gileadensis* on penile erection and fertility in mice. The gonadotropins (LH and FSH) are synthesized and secreted by the gonadotrophs of the anterior pituitary gland [28]. FSH stimulates the first division of meiosis in primary spermatocytes, resulting in secondary spermatocytes. FSH is essential for the initiation of spermatogenesis because it stimulates the production of androgen-binding protein by the Sertoli cells of the testes by binding to FSH receptors on their basolateral membranes, while LH stimulates testosterone production by Leydig cells in the testes [29]. The CGS, CGM, and CGA extracts had no effect on both hormones (FSH and LH) in diabetic mice treated with these extracts. The testosterone levels of diabetic mice treated with CGS, CGM, and CGA extracts were increased compared with the testosterone levels in untreated diabetic and normal mice. Both CGM and CGA extracts induced testosterone levels that were seven and two times higher than those of the untreated diabetic and the control groups, respectively. Notably, for the diabetic group treated with CGS, the testosterone levels exceeded those in the untreated diabetic and control groups by more than 70 and 30 times, respectively. Furthermore, the testosterone levels in the CGS group were ten times greater than testosterone levels in normal adult men. The testosterone levels in the diabetic CGS treated mice were estimated many times to verify these results. Increased testosterone levels in diabetic mice treated with different extracts of *C. gileadensis* may be independent of the hypothalamic–pituitary axis because the FSH and LH levels are unaffected by treatment with these extracts.

Erectile dysfunction is defined as the inability to attain and maintain an erection sufficient for satisfying sexual activity. The incidence of erectile dysfunction is particularly high among men with diabetes mellitus. Atherosclerosis-related vascular issues are the primary cause of organic impotence. Because penile arteries have the smallest diameter in the vascular network, erectile dysfunction may be the first sign of a widespread vascular disorder. Vascular homeostasis is largely dependent on the integrity of the endothelium. Loss of functional integrity of the endothelium and subsequent endothelial dysfunction of the first stage of atherosclerosis significantly reduces blood flow to tissues and negatively affects erectile function [30]. Homeostasis of endothelial cells is maintained in part by the synthesis of nitric oxide [31]. Nitric oxide plays a crucial role in penile erection, and the impairment of nitric oxide bioactivity has been proposed as the most significant pathological mechanism in erectile dysfunction. Adropin contributes to nitric oxide bioavailability and influences the expression of inducible NOS. Endothelial cells treated with adropin exhibit increased proliferation, migration, and formation of capillary-like tubes and decreased permeability and tumor necrosis factor-α induced apoptosis [32]. Topuz et al. assessed endothelial dysfunction and flow-mediated dilatation in patients with type 2 diabetes mellitus. The correlation between plasma adropin levels and flow-mediated dilatation values was found to be positive, and the authors proposed that adropin levels could be used to quantify endothelial dysfunction [33]. A previous study found that the adropin level was low in type 2 diabetic patients with erectile dysfunction [34]. Previously collected evidence indicates that adropin and nitric oxide are positive markers of penile erection. The present study showed that the diabetic mice treated with CGS, CGM, and CGA extracts had a higher level of both adropin and nitric oxide compared with the control group. Furthermore, different *C. gileadensis* extracts elevated nitric oxide and adropin levels in diabetic mice nearly four times higher compared to the levels in the untreated diabetic group. Overexpression of endothelin is associated with hypertension, erectile dysfunction, and heart diseases [31]. In the current study, the endothelin levels decreased in diabetic mice treated with CGS, CGM, and CGA extracts. The CGS, CGM, and CGA extracts decrease lipid peroxidation products by inducing GSH levels and antioxidant enzyme activities such as GSH-Px and SOD. The induction of positive erectile markers and reduction of the negative marker endothelin may be due to *C. gileadensis* antioxidant activity.

Regarding the semen analysis results, the sperm count was increased in diabetic mice treated with CGS. Moreover, motility and the percentage of normal morphologic sperms were raised in diabetic mice treated with CGS, CGM, and CGA extracts although not significantly. Immunohistochemistry examination of the testes showed that diabetic mice treated with *C. gileadensis* had a higher immunoreactivity of NOS compared to the control group and untreated diabetic mice. Normal testicular architecture was destroyed in the diabetic group. However, treatment with CGS, CGM, and CGA extracts restored the architecture of testicular tissue in diabetic mice and made it normal. Testosterone inhibits CD8^+^ and CD4^+^ T-cell activation. When Leydig cells were damaged, CD8^+^ and CD4^+^ T lymphocytes proliferated. During testes inflammation both CD8^+^ and CD4^+^ cells are increased [35]. Testosterone and recovery Leydig cells diminish CD8^+^ and CD4^+^ cell numbers [36]. In the current study, *C. gileadensis* reduces CD8^+^ and CD4^+^ which may decrease testes inflammation and induce its recovery in diabetic mice. Previous studies indicate that natural extracts of *Catharanthus roseus L*. have high antioxidant content that can mitigate oxidative stress in diabetic testicular tissue [37]. The authors conclude that the *C. roseus L.* maintains testicular tissue by inducing epididymal SOD and catalase*. C. gileadensis* may act similarly, potentially through pathways involving the induction of epididymal SOD and catalase. Moreover, *C. gileadensis* protects testicular tissue by its anti-inflammatory activity. The comparison between different *C. gileadensis* extracts showed that CGS had a greater effect on testosterone, nitric oxide, adropin, endothelin, semen, and immunoreactivity of testicular NOS than did CGM and CGA extracts. These differences may be due to a variation in the consistency of sap and the methanol and acetone extracts: CGS is more viscous than methanol and acetone extracts. This viscosity may reflect the fact that the CGS had a higher concentration of free radical scavengers such as saponins, flavonoids, volatile oils, sterols, and triterpenes than the methanol and acetone extracts; hence, CGS had higher antioxidant activity than the methanol and acetone extracts. Furthermore, chemical characterization of C. *gileadensis* extracts showed a high content of steroids that may induce fertility and erection in these mice [15]. Besides male infertility, polycystic ovarian syndrome (PCOS) impairs female fertility and has the same prevalence as male infertility, so focusing on PCOS treatment becomes a large scale in future studies. PCOS has emerged as the most prevalent gynecologic endocrinopathy, with a global incidence of 1.55 million patients. PCOS is defined by a range of conditions including hyperandrogenism, polycystic ovarian morphology, oligo-anovulation, hyperinsulinemia due to insulin resistance (IR), and dyslipidemia. The precise pathogenic pathways remain ambiguous; nevertheless, IR and hyperandrogenaemia are proposed to be pivotal in the pathogenesis of PCOS [38]. Hyperinsulinemia resulting from IR predominantly induces hyperandrogenism and alters steroidogenesis in both the ovaries and adrenal glands. Additionally, early sensitivity to LH inhibits follicular growth in PCOS due to modified steroidogenesis [39]. The propensity of granulosa cells to secrete anti-Müllerian hormone (AMH) results in increased serum levels of AMH. Elevated levels of AMH desensitize ovarian follicles to FSH, resulting in premature follicular arrest and impaired fertility [40].

## 5 Recommendation and conclusion

This study concludes that *C. gileadensis* raises testosterone levels and sperm count in diabetic male mice. The herb also increases positive erectile function markers (adropin and nitric oxide) and immunoreactivity of testicular NOS. Moreover, *C. gileadensis* has an anti-inflammatory effect by decreasing CD4^+^ and CD8^+^. Overall, *C. gileadensis* ameliorate erectile dysfunction and infertility in diabetic male mice due to its high steroid contents and/or its antioxidant and anti-inflammatory activities. While the present study demonstrates substantial benefits of *C. gileadensis* in diabetic mice, further research, including human clinical trials beside human cell lines, is essential to validate these findings and explore the herb’s potential for treating diabetes-induced reproductive dysfunction in clinical settings.
